# Flail chest injury—changing management and outcomes

**DOI:** 10.1007/s00068-022-02152-1

**Published:** 2022-11-01

**Authors:** Silvana F. Marasco, Jacqueline Nguyen Khuong, Mark Fitzgerald, Robyn Summerhayes, Mir Wais Sekandarzad, Vincent Varley, Ryan J. Campbell, Michael Bailey

**Affiliations:** 1 Cardiothoracic Surgery Unit, The Alfred Hospital, Melbourne, VIC 3004 Australia; 2 Department of Surgery, Monash University, Melbourne, VIC Australia; 3 National Trauma Research Institute, Melbourne, VIC Australia; 4 Trauma Service, The Alfred Hospital, Melbourne, VIC Australia; 5 Department of Anesthesiology and Perioperative Medicine, The Alfred Hospital, Melbourne, VIC Australia; 6 Australian and New Zealand Intensive Care Research Centre, Department of Epidemiology and Preventative Medicine, Monash University, Melbourne, VIC Australia

**Keywords:** Rib fixation, Chest wall injury, Rib fracture, Flail chest

## Abstract

**Purpose:**

The purpose of this study was to assess trends in management of flail chest injuries over time and to determine impact on patient outcomes.

**Methods:**

A retrospective review of data from a prospectively collated database of all trauma patients admitted to a level 1 trauma service in Victoria was conducted. All trauma patients admitted to the hospital between July 2008 and June 2020 with an Abbreviated Injury Scale (AIS) code for flail chest injury were included.

**Results:**

Our study included 720 patients, mean age was 59.5 ± 17.3 years old, and 76.5% of patients were male. Length of ICU stay decreased on average by 9 h each year. Regional anaesthesia use increased by 15% per year (0% in 2009 to 36% in 2020) (*p* < 0.001). Surgical stabilisation of rib fractures increased by 16% per year (2.9% in 2009 to 22.3% in 2020) (*p* = 0.006). The use of invasive ventilation decreased by 14% per year (70% in 2008 to 27% in 2020) (*p* < 0.001), and invasive ventilation time decreased by 8 h per year (*p* = 0.007).

**Conclusion:**

Over the past decade, we have seen increasing rates of regional anaesthesia and surgical rib fixation in the management of flail chest. This has resulted in lower requirements for and duration of invasive mechanical ventilation and intensive care unit stay but has not impacted mortality in this patient cohort.

## Introduction

Traumatic thoracic injuries account for 10% of all hospital trauma admissions and are a frequent cause of trauma-related deaths, second only to head injury [1]. Fractured ribs are present in over 20% of patients admitted to trauma centres with blunt chest trauma [2]. Flail chest represents the more severe end of the spectrum of rib fracture injury. Mortality after flail chest injury has been reported as high as 50% in the past, but a more recent nationwide survey in Canada observed a much lower mortality at 16% [3].

Flail chest is defined as the fracture of at least three consecutive ribs in more than one place, leading to a floating segment of chest wall. Flail chest can also include the sternum, whereby bilateral rib fractures on either side of the sternum lead to a floating anterior chest wall [4].

Management of these severe chest wall injuries has changed significantly over the past decade, with operative rib fixation becoming much more prevalent particularly in level 1 trauma centres [5], and other non-operative management strategies such as regional analgesia have also become more popular [6].

Our observation has been that more recent interventions such as surgical stabilization of rib fractures (SSRF) and regional analgesia have reduced some of the sequelae of flail chest injury such as need for invasive ventilation and prolonged ventilator support in the intensive care unit. These interventions are just part of a comprehensive algorithm of management aimed at reducing complications and ventilator requirements in these patients [7]. The aim of this non-comparative descriptive review was to investigate the changes in outcomes of flail chest injury over the last decade in our institution and to identify the factors that are associated with these changes.

## Methods

The Alfred Hospital data were collected from TraumaNet—a prospective database on all trauma patients admitted to the hospital. The Alfred Hospital is one of two adult level 1 trauma services in the state of Victoria, Australia. Approximately, 1200 major trauma patients are treated at The Alfred each year with an overall mortality of 8%. Approximately, 600 major chest trauma patients are treated each year with a mortality of approximately 5%. All patients admitted to the hospital between July 2008 and June 2020 with thoracic trauma and a diagnosis of multiple fractured ribs were considered for inclusion in this cohort. Patients with only a single fractured rib or who were treated for fractured ribs in the emergency department without being admitted to the hospital were not included in this group.

All patients admitted to The Alfred Hospital and coded as having a flail chest were selected for inclusion in this non-comparative retrospective analysis of prospectively collected data. Inclusion criteria were: Abbreviated Injury Scale (AIS) codes for flail chest injury. The dataset was then manually reviewed to ensure that correct coding had been used to filter the dataset. Patients were only excluded if they had been incorrectly coded and did not have a flail chest. Data on injury description, demographics, hospital interventions, and outcomes were collected.

Management of chest wall trauma and flail chest patients in our institution has evolved over the 12 years of this review with progressively more detailed algorithms and aggressive management aimed at reducing the need for invasive ventilation and reducing complications. Lower thresholds for intensive care admission, early specialist pain service assessment and management, early trial of non-invasive ventilation and thoracic surgeon assessment for rib fixation are the basic tenets of the algorithm [7].

Our technique for SSRF in these patients has been previously described [8]. We have used outer cortical plates in almost every patient (DePuy Synthes MatrixRIB [Synthes USA Products, LLC, West Chester, PA, USA] or Zimmer Biomet RibFix Blu [Zimmer GmbH, Switzerland]) with a small proportion of patients having intramedullary splints (DePuy Synthes MatrixRiB), particularly for rib fractures under the scapula.

Institutional ethics approval was granted for the study (Alfred HREC 471/20) and requirement for individual patient consent waived.

### Statistical analysis

All data were initially assessed for normality with results reported as frequency (%), mean (standard deviation) or median (interquartile range) as appropriate. Changes over time for binomial interventions and outcomes were determined using logistic regression with financial year of admission treated as a continuous variable to facilitate an annual rate of change reported as odds ratio (95% CI). Duration of ventilation and lengths of stay (hospital and ICU) were log-transformed and analysed using mixed linear regression with results reported as an annual change in percentage (95% CI). Multivariable analysis was conducted for all interventions and outcomes adjusting for age, gender, Injury Severity Score and cause of accident. All analysis was performed using SAS version 9.4 (SAS Institute Inc., Cary, NC), and a two-sided p-value of 0.05 was used to indicate statistical significance.

## Results

Over the 12 years of the retrospective review, 5865 patients with multiple fractured ribs were admitted under the trauma unit at The Alfred. Of these, 720 patients were coded as having a flail chest and these patients form the cohort for analysis in this study. Demographics of the overall group show an average age of 59.5 years (SD 17.3) (range 18–100 years), and 76.5% were male. The median Injury Severity Score (ISS) was 21 [interquartile range (IQR) 14–29], median Revised Trauma Score (RTS) 7.84 [IQR 7.55–7.84] and the median Trauma Score-Injury Severity Score (TRISS) 0.943 [IQR 0.871–0.974]. Road accidents were the most common aetiology with motor vehicle accidents (28.8%), motorcycle collisions (15.3%), bicycle accidents (6.5%) and pedestrian collisions (6.3%) contributing 56.9% of all cases. Falls were next most common contributing 30.7% of cases, with the remaining 12.4% being made up of other accidents such as horse and farming incidents.

**Fig. 1:**
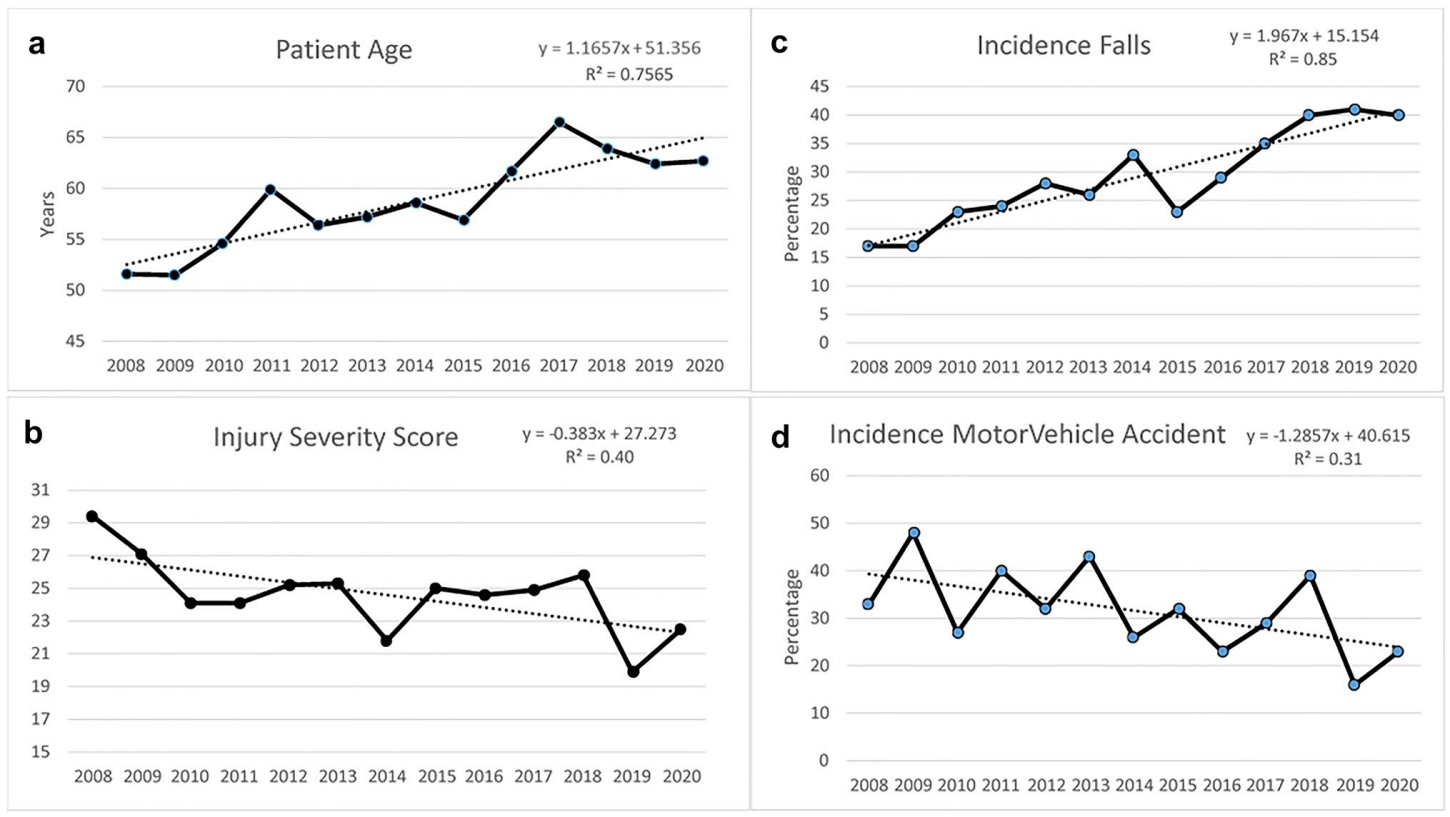
Patient Demographics. **a** Patient Age, **b** Injury Severity Score, **c** Incidence Falls, **d** Incidence Motor Vehicle Accident

A mean of 55.0 cases per year was noted (standard deviation (SD) 25.0) without any significant change in incidence over time. Analysis of the demographics over time showed that the patients admitted with flail chest were becoming progressively older over time at a rate of one year of age, per year analysed (that is, average age of 52 in 2008, vs 63 years in 2020) (Fig. 1). The mechanism of injury also changed with falls becoming a more common cause and vehicular accidents less common. The severity of injury reduced over time from an ISS median value of 27 [IQR 25–36] in 2008 to 16 [IQR 13–36] in 2020 (Fig. 1). Demographics are outlined in Table 1.

**Table 1:** Demographics—average over entire time period and analysis of change over time

Variable	Parameter	Estimate	StdErr	*R* square	*P* value
Age at injury (years) (mean [SD])	59.5 (0.64)	1.166	0.232	71.66	0.0005*
Male (%)	76.5	− 0.0078	0.0044	23.57	0.11
Cause of injury					
Motor vehicle accident	28.8% (207)	− 0.015	0.0041	55.44	0.005*
Motorcycle accident	15.3% (110)	-0.011	0.0037	45.98	0.015*
Bicycle collision	6.5% (47)	0.0016	0.0026	3.64	.055
Pedestrian	6.3% (45)	− 0.002	0.0027	5.03	0.48
Fall	30.7% (221)	0.02	0.0036	75.66	0.0002*
Struck	3.3% (24)	− 0.0005	0.0013	1.46	0.71
Horse	3.2%(23)	0.0033	0.0021	19.33	0.15
Other	6% (43)	0.0031	0.0021	18.22	0.17
Arrival at hospital observations					
Pulse (bpm)	88 [75–105]	− 0.578	0.267	31.98	0.06
Systolic blood pressure (mmHg)	139 (33.9)	− 0.097	0.387	0.63	0.81
Respiration rate (per min)	19.8 (6.57)	− 0.016	0.1	0.27	0.87
Oxygen saturation (%)	98 [95–100]	− 0.037	0.143	0.67	0.8
Inhaled oxygen fraction (%)	50.6 (29.4)	− 2.716	0.437	79.41	0.0001*
Thoracic injury details					
Pulmonary contusion (%)	32.9	0.004	0.015	0.73	0.79
Haemothorax (%)	46.4	0.046	0.012	61.69	0.002
Pneumothorax (%)	34.2	0.016	0.014	12.69	0.26
Diaphragmatic injury (%)	1.5	− 0.002	0.002	9.59	0.33
Cardiac contusion (%)	2.2	− 0.007	0.002	49.15	0.011
Glasgow coma score (GCS)					
Eye	4 [3, 4]	0.028	0.01	42.45	0.022*
Verbal	5 [4, 5]	0.032	0.012	39.68	0.028*
Motor	6 [6–6]	0.047	0.015	49.56	0.011
GCS total	15 [13–15]	0.076	0.039	27.23	0.08
ISS	21 [14–29]	− 0.429	0.189	33.85	0.047*
RTS	7.84 [7.55–7.84]	0.0064	0.0061	9.72	0.32
TRISS	0.943 [0.871–0.974]	− 0.0092	0.0026	55.41	0.005*
MAIS_head_1 (median [IQR])	0 [0–1]	− 0.044	0.019	35.23	0.042*
MAIS_face_2	0 [0-0]	− 0.0053	0.0068	5.78	0.45
MAIS_neck_3	0 [0-0]	0.012	0.0034	56.54	0.005*
MAIS_thorax_4	3 [3, 4]	− 0.038	0.016	37.64	0.034*
MAIS_abdomen_5	0 [0-0]	0.0003	0.012	0.01	0.98
MAIS_spine_6_	0 [0–2]	− 0.015	0.017	7.21	0.4
MAIS_upper_extremity_7	0 [0–2]	− 0.011	0.014	5.91	0.45
MAIS_lower_extremity_8	0 [0–1.5]	− 0.012	0.012	8.59	0.36

*ISS* injury severity score, *RTS* revised trauma score, *TRISS* trauma injury severity score, *MAIS* maximum abbreviated injury scale

Overall injury patterns are represented in the data by the system Maximum Abbreviated Injury Scale (MAIS) which for the overall cohort for thorax was a median of 3 [IQR 3–4]; head 0 [0–1]; face 0 [0-0]; neck 0 [0-0]; abdomen 0 [0-0]; spine 0[0–2]; upper extremity 0 [0–2]; lower extremity 0 [0–1.5]; burns and other trauma 0 [0-0]. Of the total cohort, 247 (33%) had some degree of lung contusion with 86 (12%) of the total cohort having major (> one lobe) contusion. Haemothorax was noted in 46% of patients and pneumothorax in 34%. Diaphragmatic injuries (at 1.5%) and cardiac contusion (at 2.2%) were relatively uncommon and noted to drop slightly in incidence over time (Table 1).

Analysis of treatment strategies revealed changes over time in many of the interventions (Table 2). Specifically, pain management changed over the years of the review with more patients receiving regional analgesia such as serratus anterior, erector spinae or.

**Table 2:** Annual change in treatment interventions

Intervention	Univariable	*P* value	Multivariable*	*P* value
	Odds ratio (95% CI)		Odds ratio (95% CI)	
Blood product transfusion	0.92 (0.81–1.04)	0.18	0.97 (0.86–1.37)	0.49
Massive transfusion protocol activated	1.04 (0.97–1.13)	0.26	1.11 (1.00–1.23)	0.06
Non-invasive ventilation	0.94 (0.85–1.04)	0.24	1.03 (0.92–1.15)	0.62
Invasive ventilation	0.84 (0.81–0.88)	< 0.0001	0.84 (0.79–0.88)	< 0.0001*
Patient-controlled/opiate infusion	0.92 (0.87–0.96)	0.0003	0.92 (0.87–0.96	0.0008*
Regional analgesia	1.17 (1.10–1.25)	< 0.0001	1.15 (1.07–1.24)	0.0002*
Epidural analgesia	1.02 (0.90–1.14)	0.79	0.97 (0.85–1.11)	0.67
Intercostal catheter pleural drainage	0.98 (0.93–1.03)	0.35	0.97 (0.91–1.03)	0.27
Surgical stabilisation rib fractures	1.12 (1.06–1.19)	0.0001	1.15 (1.07–1.24)	0.0001*
Tracheostomy	0.86 (0.79–0.93)	0.0003	0.84 (0.75–0.93)	0.0015*

**Fig. 2:**
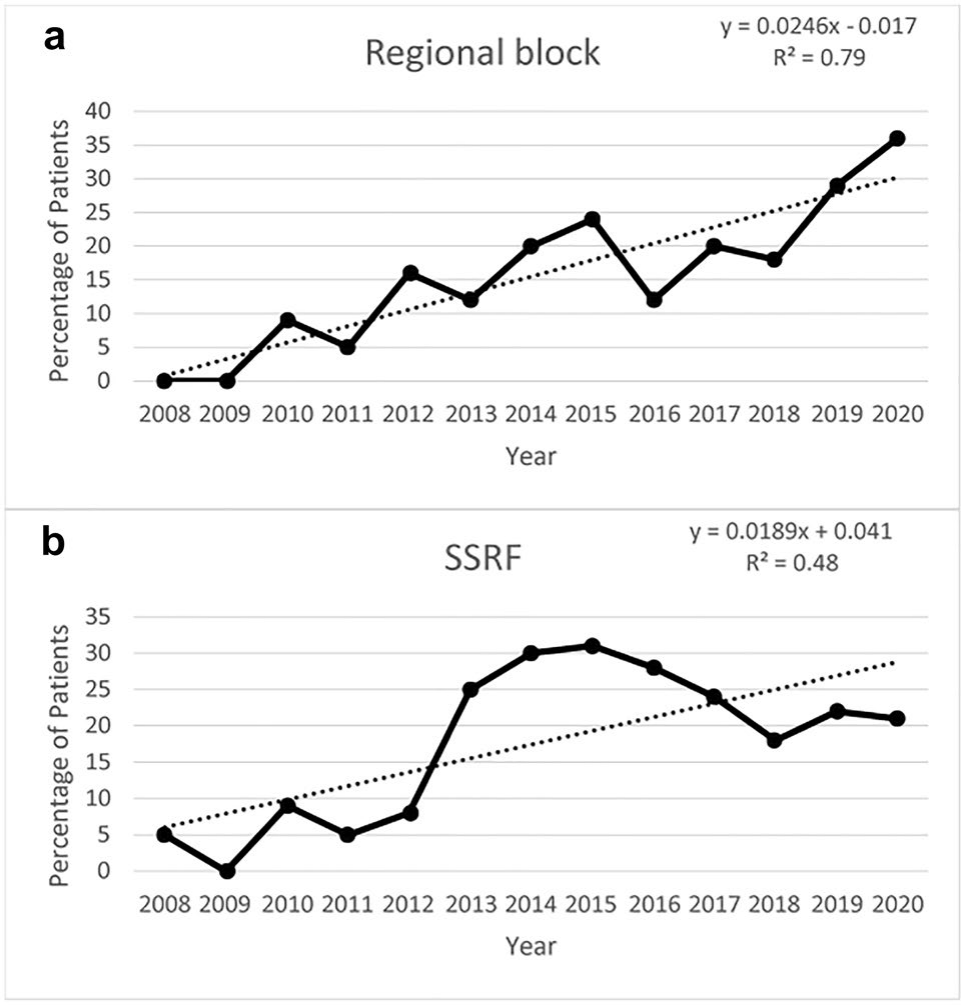
Interventions. **a** Regional Block, **b** Surgical Stabilisation of Rib Fractures (SSRF)

**Fig. 3:**
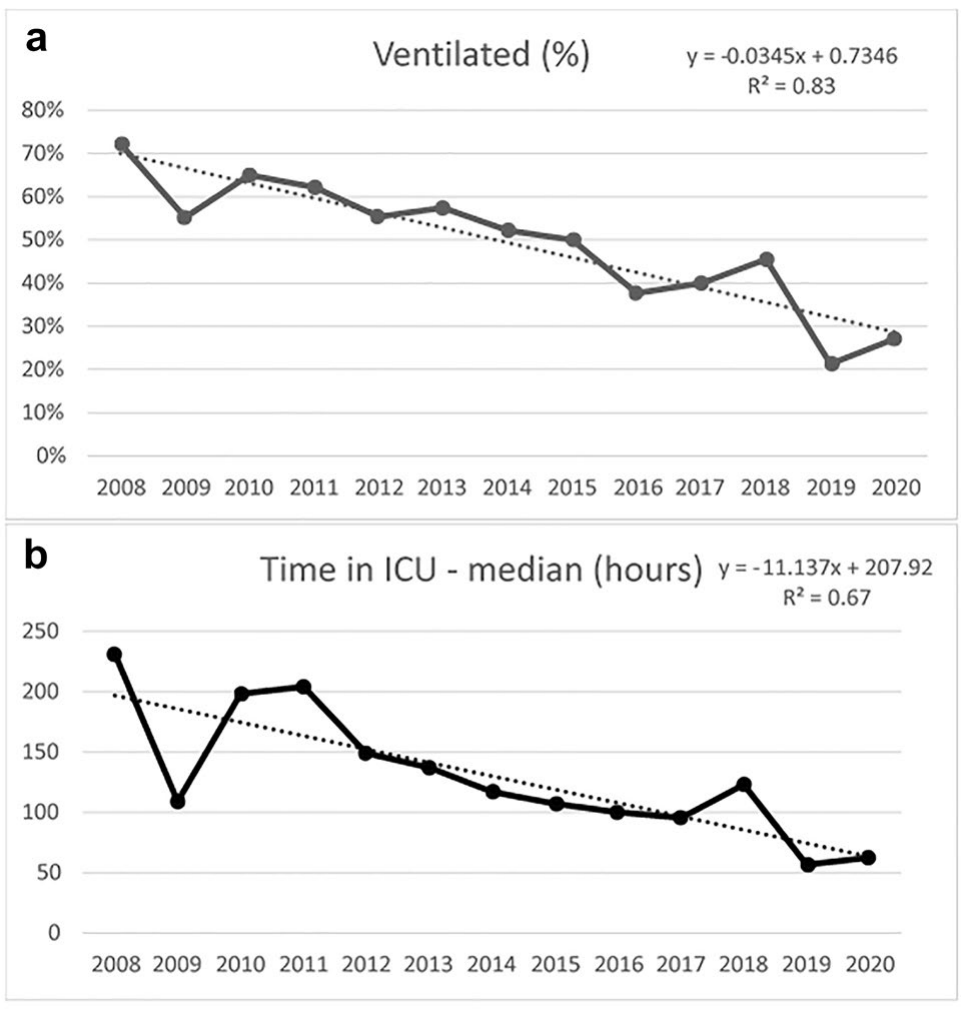
Outcomes. **a** Invasive ventilation—percentage of patients, **b** Time in the intensive care unit (hours)

*Adjusted for age, gender, Injury Severity Score, haemothorax, pneumothorax, pulmonary contusion, and cause of accident

paravertebral continuous catheter infusions (increasing at a relative rate of 15% per year) (Fig. 2). There was a corresponding relative decrease of 18% per year in patients being treated with patient controlled or continuous opiate infusions. Surgical stabilisation of rib fractures (SSRF) also increased over the decade at a relative rate of 16% per year (Fig. 2). In total, 137 flail chest patients underwent SSRF over the 12 years. Ventilatory support with invasive ventilation decreased at a relative rate of 14% per year (Fig. 3), and tracheostomy insertion decreased at a relative rate of 17% per year. Multivariable analysis of treatment interventions, adjusted for age, gender, ISS, haemothorax, pneumothorax, pulmonary contusion and cause of accident, showed that these significant changes over time remained (Table 2).

Further analysis was performed to determine if changes in management were responsible for the reduction in invasive ventilation hours over time. Logistic regression was performed with invasive ventilation as the outcome and age, gender, ISS, cause of injury, haemothorax, pneumothorax, pulmonary contusion, SSRF and regional analgesia as predictors. As can be seen in Table 3, the key predictors for invasive ventilation were ISS, presence of pneumothorax, haemothorax, SSRF and year. This indicates that the decline over time for ventilation is not driven by age, ISS, SSRF or regional analgesia. Patients who underwent SSRF were four times more likely to be supported with invasive ventilation. Analysis of the SSRF patients showed no significant differences in age between the SSRF and no SSRF cohorts (mean 58.7 ± 16.7 vs 59.7 ± 17.3 years; p = 0.65) but a significantly higher ISS score in the SSRF patients vs no SSRF (median 25 [IQR 17–36] vs 20 [IQR 13–29]; *p* < 0.0001). SSRF patients were also more likely to have sustained their rib fractures in a motor vehicle accident (52 vs 41%; *p* = 0.049). Patients who underwent SSRF were four times more likely to be supported with invasive ventilation. Of the patients who underwent SSRF, 50/137 (36.5%) were intubated on arrival to hospital and a further 24/137 (17.5%) required intubation prior to SSRF. In contrast, of the patients who did not proceed to SSRF, 184/569 (32%) were intubated prior to arrival in hospital and another 48/569 (8.4%) were intubated after arrival in hospital. Overall, SSRF patients were more likely to be intubated for their injuries (74/137 (54%)) than non-SSRF patients (232/569 (41%)); *p* = 0.005. SSRF patients who were intubated pre-operatively required ventilatory support for a median of 100 h (IQR 77–155) prior to SSRF and a median of 21 h (IQR 0–84) post-operatively.

**Table 3:** Logistic regression analysis of invasive ventilation

Effect	Odds ratio	*P* value
Age (years)	0.99 (0.98–1.01)	0.22
Cause		0.10
Cause fall vs struck	0.85 (0.31–2.37)	
Cause horse vs struck	1.14 (0.29–4.49)	
Cause motor vs struck	2 (0.73–5.51)	
Cause motorcycle vs struck	1.05 (0.38–2.93)	
Cause other vs struck	1.53 (0.48–4.93)	
Cause pedal vs struck	1.14 (0.36–3.61)	
Cause pedestrian vs struck	1.69 (0.52–5.56)	
Year	0.84 (0.79–0.88)	< 0.001*
ISS	1.08 (1.06–1.09)	< 0.001*
Male	1552 (0.99–2.43)	0.06
Regional analgesia	0.94 (0.56–1.56)	0.80
Thoracic injury		
Pulmonary contusion	1.02 (0.7–1.49)	0.91
Haemothorax	1.59 (1.08–2.35)	0.02
Pneumothorax	1.93 (1.29–2.9)	0.001
Diaphragmatic injury	1.08 (0.25–4.68)	0.92
Cardiac contusion	1.04 (0.35–3.1)	0.94
SSRF	4.06 (2.43–6.76)	< 0.001*

*ISS* injury severity score, *SSRF* surgical stabilisation of rib fractures

In terms of the relationship with SSRF, patients with pulmonary contusion were twice as likely to undergo SSRF (OR 2.05 [1.32–3.17]; *p* = 0.0013); patients with haemothorax were three times more likely to undergo SSRF (OR 2.93 [1.83–4.69]; *p* < 0.0001), and these relationships did not change over the decade of the study.

Procedures performed simultaneously with the SSRF included thoracoscopy in only seven patients and bronchoscopy in three. Six patients required lung repair which was performed through the SSRF wound using suture repair. Almost all patients have the pleural space opened during the SSRF to allow evacuation of retained haemothorax, and intercostal catheter is inserted in every SSRF patient. We also place a smaller drain in the chest wall muscle layers for 48 h to prevent haematoma formation over the plates. SSRF was performed a median of 5 days post-injury (range 1–17 days), and this did not change significantly over the 12 years of the study.

Complications such as pneumonia occurred in 29% of all patients, sepsis in 9.4% and adult respiratory distress syndrome in 1.6%. None of these complications changed in incidence over time. Multivariable analysis of pneumonia found that patients undergoing SSRF were 2.7 times more likely to develop pneumonia during their admission (OR 2.72 [1.69–4.38]; *p* < 0.0001); however, we do not have information in our database on whether this developed before or after SSRF.

Multivariable analysis of outcomes adjusted for age, gender, ISS and cause of accident showed significant decreases over time in ICU length of stay, hospital length of stay and duration of invasive ventilation (Table 4).

**Table 4:** Annual change in outcomes

Outcome	Univariable		Multivariable*	
	Odds ratio (95% CI)	*P* value	Odds ratio (95% CI)	*P* value
Admission to ICU	0.98 (0.94–1.03)	0.45	1.01 (0.96–1.06)	0.76
ARDS	1.10 (0.90–1.33)	0.35	1.10 (0.88–1.37)	0.40
Hospital mortality	1.04 (0.97–1.13)	0.26	1.05 (0.97–1.15)	0.24
Discharge home	1.02 (0.97–1.06)	0.50	1.01 (0.96–1.06)	0.74
Discharge other	1.01 (0.93–1.09)	0.88	0.97 (0.89–1.06)	0.49
Discharge rehabilitation	0.97 (0.93–1.01)	0.16	0.97 (0.92–1.02)	0.20
Pneumonia	0.95 (0.91–1.00)	0.07	0.96 (0.91–1.02)	0.17
Sepsis	0.99 (0.92–1.07)	0.84	1.00 (0.92–1.09)	0.99

	Annual % change (95% CI)		Annual % change (95% CI)	
ICU length of stay	− 0.07 (− 0.10 to − 0.05)	< 0.0001	− 0.06 (− 0.08 to − 0.03)	< 0.0001*
Duration of ventilation	− 0.03 (− 0.06 to 0.01)	0.15	− 0.04 (− 0.08 to − 0.01)	0.02*
Hospital length of stay	− 0.02 (− 0.05 to 0)	0.07	− 0.02 (− 0.04 to 0.01)	0.20

*ARDS* adult respiratory distress syndrome, *ICU* intensive care unit

Admission to the intensive care unit (ICU) remained static over the period of the study with a mean of 73% of patients requiring ICU admission. However, the length of stay in ICU dropped by approximately 9 h per year (Fig. 3).

Overall mortality was 9.2%, and this did not change significantly from year to year. Forty-one percent of patients were discharged home, and 43% were discharged to a rehabilitation hospital. The remaining 7% of patients were discharged to another care facility.

Of the 66 mortalities, 18 were due to traumatic brain injury, 22 were from comorbidities, 16 due to sepsis, and the remaining 10 were due to other causes or not adequately coded.

## Discussion

In this study, we have identified changes in the demographics of the patients presenting with flail chest to our institution over the last 12 years, as well as significant changes in the management of these patients in our institution. We are seeing older patients, with a greater incidence of falls as the mechanism of injury rather than motor vehicle accidents. This is likely to explain the lower ISS seen over time as patients are less likely to have multi-system injuries after a fall compared to a motor vehicle accident.

Multi-modality management has included an increasing use of regional analgesia catheter techniques and SSRF which has coincided with fewer patients requiring invasive ventilation and a shorter duration of invasive ventilation. However, multivariable analysis was not able to identify a causative factor for the decrease in invasive ventilation requirement or hours. We had postulated that our management strategies, particularly SSRF and regional analgesia techniques would be the cause of reduced ventilation requirement and earlier ICU discharge. However, we were not able to demonstrate this statistically. It is likely that a combination of factors, including our interventions mentioned above, as well as the progressive implementation of a comprehensive chest trauma management pathway for these patients has contributed [7]. These management algorithms have included lower thresholds for admission to ICU which are likely to allow earlier discharge of less sick patients and thus account for shorter ICU stay.

The use of SSRF has increased substantially in our institution over the timeline of this study. In 2008, we were just commencing SSRF and were introducing it first with a pilot study of only a dozen patients and then followed with a randomised controlled trial of SSRF versus non-operative management which was run by the thoracic surgeons introducing the procedure [9, 10]. After that study was published, SSRF rates increased markedly with significant support from all stakeholders such as the trauma unit, physiotherapists, intensivists and anaesthetists. The final decision to offer SSRF is made by the thoracic surgeons after consultation with the referring teams and follows the current evidence and expert consensus reports available in the literature [11]

Traumatic flail chest injury has been associated with a mortality rate of 33–80% quoted in early literature [12–14]; however, mortality rates have reduced substantially in more contemporary cohorts of national registry data with 20.6% quoted in a study of 262 flail chest patients in Israel [15] and 16% quoted in a Canadian study of 3467 patients [3]. Our overall mortality was 9.2%, and this did not change appreciably over the 12 years of the study.

The most common cause of flail chest injury historically is motor vehicle accident, accounting for 79% [3] and 76% [15] in registry studies. We saw a much lower incidence of 29% overall, and this had decreased substantially over the decade in our cohort from 40% in 2009 to 21% in 2020. There was a corresponding increase in falls being a major cause from 20% in 2009 to 38% in 2020. The changes in aetiology seen may be due to a number of factors. One of those is more stringent hospital coding with the inclusion of radiological flail segments which may not be as significant clinically. This would also account for the lower ISS seen over the decade and may partly explain the lower mortality rate in our cohort. Another reason for a reduction in road trauma as a major cause is the recent COVID-19 pandemic which has seen a marked reduction in road traffic with repeated lockdowns in many countries. In fact, Melbourne, Australia, the site of this study had an unprecedented 260 days of lockdown over 2020–2021. Despite this, the reducing impact of road trauma predated the pandemic with a steady drop in road trauma as a cause of flail chest injury seen over the decade.

The high mortality rates reported in flail chest injury reflect the high impact nature of the injury as well as associated life-threatening injuries. In other registry series, severe head injury is seen in 15 – 27%, pulmonary contusion in 54%, and haemothorax, liver lacerations and splenic lacerations are also commonly seen [3, 14]. Mortality risk has been shown in several studies to be increased with age ≥ 65 years, associated brain injury, and bilateral flail chest injury [16]. Our cohort did not have a high incidence of major injury in other body systems, and the mortality was lower than other reported series, at 9.2%. Despite this, we have not been able to reduce our mortality rate with our changes in management over time. Furthermore, our patients have had significantly lower ISS over time, and we have still not seen a mortality difference.

Patients with flail chest often require invasive mechanical ventilation and prolonged intensive care unit stay. As a result, there is an increased risk of complications such as tracheostomy, pneumonia, sepsis and adult respiratory distress syndrome (ARDS) [3]. Although we saw a reduced requirement for and duration of invasive ventilation, we did not see any changes in the requirement for non-invasive ventilatory support or in the incidence of complications of sepsis, pneumonia or ARDS.

We performed SSRF in our patients a median of 5 days post-injury. This could have impacted on outcomes, as early SSRF (within 3 days of admission) has been associated with shorter hospital and intensive length of stay, shorter duration of mechanical ventilation, and lower rates of pneumonia and tracheostomy [17]. Although we aim to perform SSRF as soon as practical, logistical constraints often delay progress to operative intervention and we have not improved on this over the 12 years of this study.

Analgesia is a critical component of the management of these patients. Without adequate pain relief, it is unlikely that physiotherapy and non-invasive ventilatory support will be successful. Analgesic protocols include an escalating regime of oral or parenteral analgesia depending on the condition of the patient and the extent of injury. Typically, these include regular acetaminophen, with a COX-2 inhibitor and a demand-only opioid such as fentanyl or morphine [18]. However, systemic based analgesia has its limitations particularly given the alterations in pharmacokinetic and pharmacodynamic effects of opioids on the elderly and frail patients with multisystemic disease can lead to opioid induced ventilatory inhibition and sedation.

Regional analgesia is being used increasingly in those patients in whom oral and parenteral therapy do not provide sufficient analgesia, and in those patients deemed to be at higher risk of pulmonary complications. These continuous catheter infusion techniques are increasingly focussed on myofascial plane blocks such as serratus anterior blocks and erector spinae blocks [19]. Benefits in the elderly, particularly in opioid sparing and reduction of delirium, have been demonstrated [20]. Interestingly, that study found no impact on mortality, or respiratory complications in a cohort of patients greater than 65 years. In recent years, a focus towards ultrasound-guided regional anaesthesia techniques and a move away from systemic-based analgesia or traditional regional techniques (such as thoracic epidural analgesia and/or thoracic paravertebral catheters) has been emerging as a trend in major trauma centres, and is very much reflected in our results here.

SSRF in ventilator dependent flail chest patients has been shown in multiple studies to reduce ventilator time and ICU stay. Three randomised controlled trials comparing SSRF to non-operative management have shown reductions in ventilator duration and ICU stay [9, 21, 22].

Since then, a number of meta-analyses have combined the published outcomes of almost 6000 patients (although less than 1300 patients received operative fixation). These analyses are consistent in their findings that in flail chest patients, operative fixation offers benefits to mortality, hospital length of stay, duration of mechanical ventilation, incidence of pneumonia, and requirement for tracheostomy [23].

## Conclusion

Over the past decade, we have seen increasing rates of regional anaesthesia and surgical rib fixation in the management of flail chest. We have also seen lower requirements for and duration of invasive mechanical ventilation and intensive care unit stay in flail chest patients over the last decade, although mortality rates have not appreciably changed over this time. Our progressive implementation of a comprehensive chest trauma pathway has allowed more streamlined management of these patients.
